# Revisiting the three-factor model of eating disorders: the role of self-compassion

**DOI:** 10.1186/2050-2974-1-S1-O26

**Published:** 2013-11-14

**Authors:** Tracey Wade, Jane Cooper, Deb Rattray

**Affiliations:** 1 School of Psychology, Flinders University, Australia

## 

Three-factor model posits that a three-way interaction between weight concern, perfectionism, and self-efficacy predict changes in disordered eating but has proved difficult to replicate. The aim of this study was to investigate a revised model, examining a three-way interaction between concerns, perfectionism, and self-compassion in predicting changes in disordered eating. Women (N=55) with a mean age of 23.89 years completed questionnaires on two occasions, 4 to 6 months apart. The three-way interaction was significant, as was the contribution of self-compassion, the concern score from the Eating Disorder Examination, and all of the 2-way interaction terms. The whole model accounted for 43% of the variance of the change in disordered eating. Women experienced decreases in disordered eating if they had high level of concerns but low levels of perfectionism and high levels of self-compassion. This suggests that interventions that tackle both perfectionism and self-compassion may be useful in the prevention and treatment of eating disorders. Subsequent examination of a short self-compassion intervention confirmed that, compared to a distraction and control condition, it increased weight satisfaction. Additionally, in comparison to the control condition, it decreased belief in perfectionistic thinking.

This abstract was presented in the **Disordered Eating – Characteristics & Treatment** stream of the 2013 ANZAED Conference.

